# Cross-cultural adaptation and psychometric evaluation of the Spanish BEDS-7 in a high-symptom Chilean sample: evidence for a two-factor model

**DOI:** 10.1186/s40337-026-01623-9

**Published:** 2026-05-02

**Authors:** Neli Escandón-Nagel, Mauro P. Olivera, Carlos Baeza-Henríquez, Lea Vallejos-Barrera, Eva Trujillo-ChiVacuan

**Affiliations:** 1 Departamento de Psicología, Facultad de Ciencias de la Salud, Universidad Católica de Temuco, Temuco, Chile; 2 Escuela de Psicología, Facultad de Ciencias Sociales y Educación, Universidad Santo Tomás, Temuco, Chile; 3 Departamento de Psicología, Facultad de Ciencias de la Educación, Ciencias Sociales y Humanidades, Universidad de La Frontera, Temuco, Chile; 4 Research Department, Comenzar de Nuevo, Monterrey, México; 5 Tecnológico de Monterrey, Escuela de Medicina y Ciencias de la Salud, Monterrey, México

**Keywords:** Binge eating disorder, Psychometric evaluation, Spanish-language adaptation, Measurement invariance, Screening instrument

## Abstract

**Background:**

Binge Eating Disorder (BED) is prevalent among adults with overweight/obesity, yet its detection remains limited by the scarcity of validated tools for Spanish-speaking populations. This study aimed to culturally adapt and evaluate the psychometric properties of the Binge Eating Disorder Screener-7 (BEDS-7) in Chilean adults with overweight/obesity and recurrent binge-eating episodes.

**Methods:**

The BEDS-7 was translated, back-translated, reviewed by expert judges, and pilot tested to ensure linguistic and cultural adequacy. Content validity evaluation also led to the exploratory addition of two DSM-5–based items. In total, 1,497 adults were recruited; after applying eligibility criteria, 435 were retained. Participants completed the Spanish BEDS-7 and online measures. Content validity was evaluated through expert judgment, structural validity using confirmatory factor analysis and exploratory structural equation modeling, internal consistency using omega coefficients, measurement invariance using multigroup analyses across gender, BMI, and physical activity, and concurrent validity through associations with negative affect.

**Results:**

Content validity analyses indicated adequate clarity, coherence, and relevance, although lower sufficiency supported the exploratory inclusion of two additional items. For the BEDS-7, a two-factor structure showed excellent fit (CFI = 0.995; TLI = 0.998; RMSEA = 0.014) and acceptable internal consistency (ω = 0.706), with factors labeled “loss of control” and “emotional distress.” Measurement invariance was supported across gender, BMI, and physical activity groups, and negative affect was positively associated with both factors.

**Conclusions:**

The Spanish BEDS-7 demonstrated adequate psychometric properties for assessing binge-eating symptomatology in Chilean adults with overweight/obesity and recurrent binge-eating. This study provides novel evidence from Latin America and contributes to cross-cultural comparability. Future research should assess diagnostic accuracy and temporal stability.

## Abstract (plain language summary)

Binge-eating involves eating large amounts of food while feeling unable to stop. It is common among adults living with overweight or obesity, but many people are not identified early because very few short screening tools exist in Spanish. To help address this gap, we adapted the Binge Eating Disorder Screener-7 (BEDS-7) for use in Chile. Nearly 1,500 adults took part, and 435 met the criteria needed for the analysis. We first checked whether the questions were clear and meaningful for Chilean adults, which led to the addition of two items. We then examined how the questions grouped together and found two main areas: loss of control around eating and emotional distress related to eating. The questionnaire also worked in a similar way for people of different genders, different body-weight categories, and different levels of physical activity. These findings suggest that the Spanish BEDS-7 can be a useful tool for assessing binge-eating symptoms in Chilean adults with overweight or obesity and recurrent binge-eating.

## Background

Binge Eating Disorder (BED) is defined in the Diagnostic and Statistical Manual of Mental Disorders, Fifth Edition (DSM-5) [1] by recurrent episodes of excessive intake within a short period with loss of control, occurring at least once weekly for three months. Globally, BED is the most prevalent eating disorder—0.6–1.8% in adult women and 0.3–0.7% in adult men—exceeding anorexia nervosa and bulimia nervosa [2], underscoring its public-health relevance. In Latin America, epidemiological data on BED remain limited [3], although available evidence indicates that the disorder is associated with psychiatric comorbidity, functional impairment, and reduced quality of life [4].

A study conducted in the United States found that binge-eating behaviors were more common among individuals with obesity, and that those who binge were more likely to have obesity, although this association does not imply causation [5]. Importantly, binge-eating symptomatology is strongly associated with overweight and obesity, making this a clinically relevant population in which the impact of binge eating may be particularly significant [3], and supporting its selection as the focus of the present study. BED also frequently co-occurs with mood, anxiety, and substance use disorders [6]. Psychiatric comorbidities have been linked to higher Body Mass Index (BMI) trajectories [7], greater depression in individuals with overweight or obesity [5], and more dysfunctional personality traits among those with obesity who binge compared to those who do not [8].

In Spain, Escandón-Nagel et al. [9] found that individuals with obesity and BED reported higher emotional eating and greater eating-related emotional conflict than those with obesity without BED. More recently, Escandón-Nagel et al. [10] showed that BED in individuals with obesity is associated with more pronounced eating-related symptomatology and elevated emotional distress. Together, these findings highlight the significant emotional burden linked to BED in obesity and reinforce the importance of having accurate and culturally adapted assessment instruments. Despite this impact, many individuals with BED remain undiagnosed. Cossrow et al. [11] showed significant diagnostic delay and limited recognition of the disorder in the United States, challenges that persist in many Spanish-speaking countries where there is a notable scarcity of brief, cross-culturally adapted and validated instruments for BED.

Given these diagnostic challenges, several instruments have been used to assess binge-eating symptoms. The Eating Disorder Examination Questionnaire (EDE-Q) [12] is widely used but is not BED-specific and is relatively lengthy (28 items) [13]. The Binge Eating Scale (BES) [14] evaluates severity and emotional/behavioral correlates of binge episodes, but it was not designed as a screening tool, limiting its utility for early detection.

The 7-item Binge Eating Disorder Screener (BEDS-7) [15] was specifically developed to identify individuals at risk for BED. Its brevity enables rapid administration, and previous studies have reported favorable sensitivity for identifying individuals with BED symptomatology [15]. Grounded in DSM-5 criteria, it ensures consistency in professional communication and is particularly valuable in clinical practice, helping professionals identify and address BED with patients [16]. However, its performance and interpretation may vary depending on the population and context in which it is applied, underscoring the need for context-specific psychometric evaluation. In Chile, the BEDS-7 could thus serve as a key tool for both clinical and epidemiological purposes, offering a practical response to local and regional challenges in the identification of binge-eating symptomatology. There is limited evidence about the content coverage of the BEDS-7 across cultural settings. According to validity assessment guidelines, item content should be systematically evaluated to ensure conceptual representativeness of each construct domain [17]. Therefore, analyzing content sufficiency was considered a key component of the adaptation process.

A recent study provided preliminary evidence of the reliability and validity of the BEDS-7 in a cross-cultural sample comprising 42 countries, including Chile [18]. In that study, a unidimensional model was tested and demonstrated acceptable fit indices in the confirmatory factor analysis conducted on the global sample, representing a relevant step forward in the cross-cultural evaluation of the instrument. However, the study did not report any linguistic or cultural adaptation process specific to each country, nor did it include psychometric analyses focused on clinical subgroups [18].

In the case of Chile, only descriptive values were reported; no psychometric properties were presented. Within this study, Chile showed relatively elevated BEDS-7 scores compared to other countries, representing one of the few available references for understanding binge-eating symptomatology in this context.

Considering the above, the present study aims to evaluate selected measurement properties of the BEDS-7 in a sample of Chilean adults with overweight or obesity and recurrent binge-eating episodes, including content validity, internal structure, internal consistency, measurement invariance, and concurrent validity. To achieve these aims, the study was conducted in two sequential and methodologically linked phases: an initial phase of linguistic adaptation and content validation, followed by a second phase of psychometric evaluation in a clinically enriched sample. Based on prior literature, we formulated the following hypotheses: (1) the adapted BEDS-7 would demonstrate adequate content validity; (2) a factorial structure with acceptable to excellent fit indices would emerge; (3) the scale would show measurement invariance across gender, nutritional status, and physical activity; and (4) higher negative affect would be associated with greater binge-eating symptomatology. By providing one of the first country-specific validation efforts of the BEDS-7 in Chile, this study supports cross-cultural comparability and contributes to improving the assessment of binge-eating symptomatology in diverse populations.

## Method

This study is part of the project funded by the National Research and Development Agency (ANID), through the Fondecyt Iniciación Grant No. 11230678.

Reporting of the study prioritized transparency in participant recruitment, selection, and analytic procedures, in line with recommendations for reporting diagnostic studies where applicable (STARD 2015; [19]).

In addition, the evaluation of measurement properties was guided by selected COSMIN recommendations [20], in accordance with the scope and aims of the present study.

### Design

This is a quantitative, non-experimental, cross-sectional study with a descriptive-instrumental scope. The study was conducted in two sequential and methodologically linked phases. Phase 1 involved the linguistic adaptation and content validation of the BEDS-7 through expert review and pilot testing, with the purpose of generating a linguistically appropriate and contextually relevant version of the instrument. This adapted version constituted the basis for Phase 2, which focused on the psychometric evaluation of the instrument through its administration to a large sample of participants.

### Phase 1: linguistic adaptation and content validation by expert judges

#### Participants

Expert Judges: Seven expert judges participated in this phase. All were trained in clinical psychology and research, with experience in the field of eating disorders.

Pilot Sample: In October 2023, a convenience sample of 10 adults was recruited. Sociodemographic and clinical characteristics are presented in Table 1.

Inclusion criteria were: (a) residing in Chile, (b) being over 18 years of age, (c) having experienced at least two binge eating episodes in the past 30 days, and (d) presenting with overweight or obesity.

Exclusion criteria were: (a) pregnancy or breastfeeding for ≤ 6 months, (b) self-induced vomiting as a compensatory strategy for weight or shape control, and (c) a history of bariatric surgery for weight loss.

**Table 1:** Sociodemographic and clinical characteristics of the pilot sample (*N* = 10)

Variables	Descriptives
Age (SD)	34 (8.54)
BMI (SD)	30.75 (3.56)
Number of binge episodes (SD), range	5.60 (3.34) (2–11)
Gender (%)	
Female	80%
Male	20%
Socioeconomic level (%)	
High	-
Upper-middle	10%
Middle	40%
Lower-middle	50%
Low	-
Engages in physical activity	
Yes	40%
No	60%

#### Instruments

- Expert Judgment Form: An ad hoc form was developed to assess the content validity of the BEDS-7. It included the construct definition and target population, and asked judges to evaluate each item for clarity, coherence, and relevance (yes/no). A global assessment of clarity, coherence, relevance, and sufficiency was then provided using a 5-point Likert scale, with space for additional comments.
- Binge Eating Disorder Screener-7 (BEDS-7, 15): This 7-item instrument includes two dichotomous items assessing the presence of binge-eating episodes and associated distress (items 1–2), and five items reflecting DSM-5 (1) diagnostic criteria for BED (items 3–7), rated on a 4-point Likert scale (0 = Never or rarely to 3 = Always). The original scoring algorithm classified individuals as high risk if they endorsed items 1–2, scored 1–3 on items 3–6, and 0–1 on item 7 (sensitivity = 100%, specificity = 38.7%) [13]. More recent studies have used a summed total score (0–17), coding items 1–2 as 0 = No and 1 = Yes [18, 21–22].
- Written Cognitive Feedback Instrument: An ad hoc questionnaire was developed to assess participants’ understanding of the BEDS-9 (BEDS-7 plus two items added after expert review; see Results). It included four open-ended questions about confusing or difficult items, sources of difficulty, and suggestions for improvement. The questionnaire was used to identify ambiguities and evaluate the cultural and linguistic adequacy of the BEDS-9 in Chile.
- Sociodemographic Questionnaire: Developed by the research team, it included items on age, gender, socioeconomic status, physical activity, self-reported weight and height used to calculate body mass index (BMI), and binge-eating episodes in the past month. Binge-eating episodes were assessed using two self-report items referring to the last 30 days: one asking whether the participant had experienced any episode of eating excessively accompanied by a sense of loss of control, and another asking for the number of such episodes. These binge-eating items were reviewed by expert judges during Phase 1 to ensure clarity and content adequacy.

#### Procedure

The BEDS-7 was translated into Spanish by two professional translators and back-translated by a bilingual psychologist. Expert judges reviewed the version and suggested minor modifications (see Results). For pilot testing, participants were recruited through social media, posters, and healthcare referrals. Eligibility was verified by phone, and those who qualified provided informed consent and completed the online questionnaires (SurveyMonkey). Participants received compensation of 10,000 Chilean pesos (approximately USD 10). The study was approved by the Ethics Committee of the Universidad Católica de Temuco. No major comprehension issues were reported.

#### Data analysis plan

To assess content validity, Aiken’s V coefficient was calculated [23] using a significance level of *p* = .05. This index estimates expert agreement on item relevance (0–1 range), with higher values indicating greater content validity. As the number of judges increases, the acceptable minimum value for Aiken’s V may be slightly lower, and confidence intervals become narrower. In this study, values ≥ 0.70 with confidence intervals between 0.62 and 0.90 were considered acceptable, following previous recommendations [24–25].

Aiken’s V and its confidence intervals were computed using the equations proposed by Penfield and Giacobbi [26], which provide more robust interval estimates than the original method.

### Phase 2: psychometric evaluation

#### Participants

**Fig. 1:**
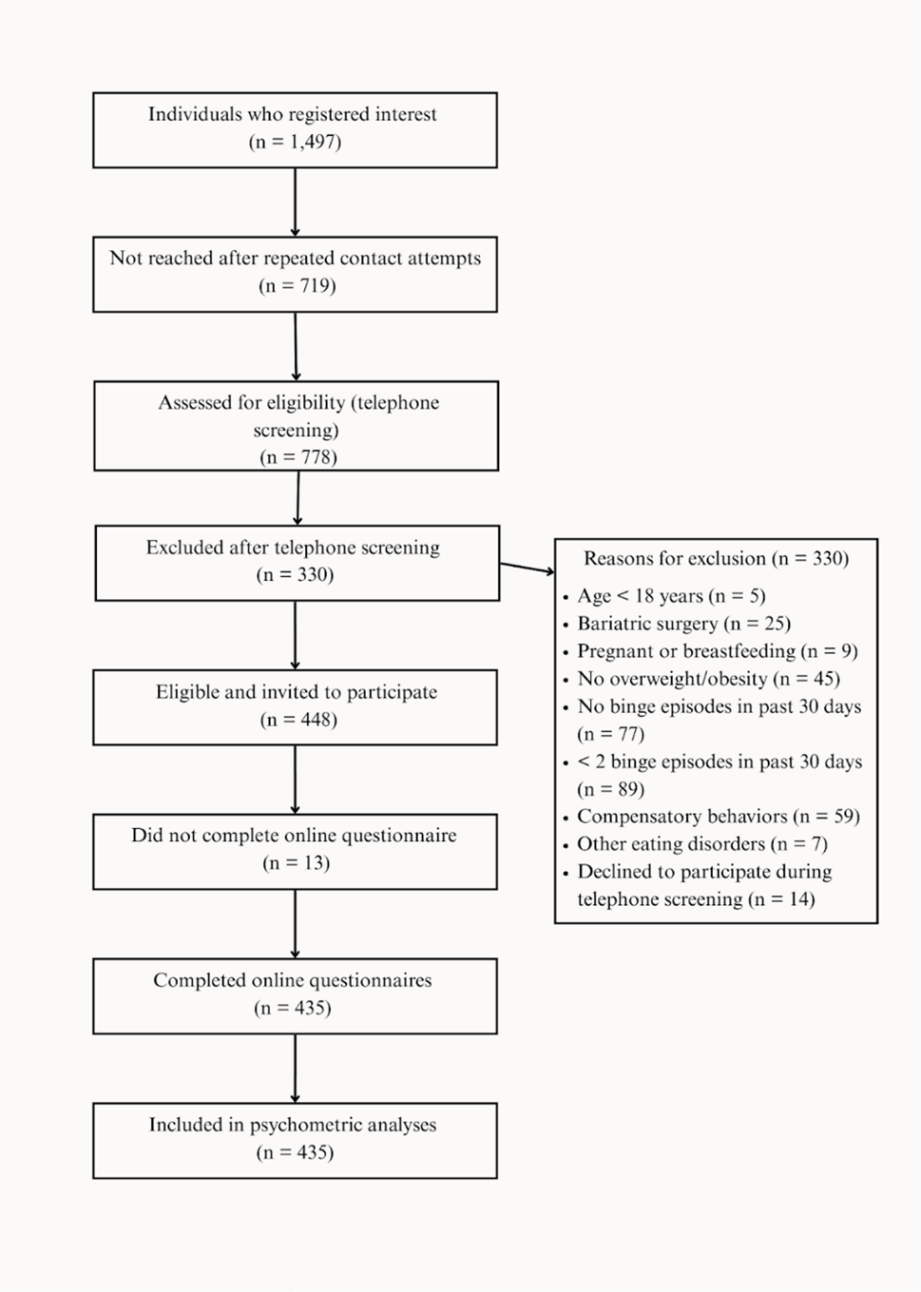
Participant flow diagram showing recruitment, telephone screening, eligibility assessment, and inclusion in psychometric analyses

A total of 1,497 individuals registered interest in the study. After eligibility screening and application of predefined inclusion and exclusion criteria, the final analytical sample comprised 435 participants. A detailed description of the recruitment, screening, and selection process is provided in the Procedure section and illustrated in Fig. 1.

The final sample size (*N* = 435) was considered adequate for psychometric analyses. The subject-to-item ratio (62:1) exceeded recommended guidelines. In addition, simulation studies indicate that samples of 200–300 are sufficient for models of similar complexity (i.e., fewer than 10 items, two correlated factors, and factor loadings above 0.50) [27]. A post hoc power analysis (pwrSEM) further supported adequate statistical power (> 0.80) to detect model misfit [28].

Data were collected through non-probability convenience sampling between October 2023 and February 2025. Of the participants, 69.4% identified as female, 28.3% as male, 1.8% as non-binary, 0.2% as “other,” and 0.2% as “prefer not to say.” Gender-based analyses included only female and male participants. The mean age was 33.25 years (SD = 9.41; range = 18–61). Descriptive statistics are presented in Table 2.

**Table 2:** Sociodemographic and clinical characteristics of the final sample (*N* = 435)

Variables	Descriptives
Age (SD)	33.25 (9.41)
BMI (SD)	33.44 (6.44)
Nutritional status	
Overweight	33.9%
Obesity	66.1%
Number of binge episodes (SD), range	7.58 (5.73) (2–35)
Gender (%)	
Female	69.4%
Male	28.3%
Non-binary	1.8%
Prefer not to say	0.2%
Socioeconomic level (%)	
High	-
Upper-middle	5.1%
Middle	45.1%
Lower-middle	34%
Low	15.9%
Engages in physical activity	
Yes	45.1%
No	54.9%

#### Instruments

- Binge Eating Disorder Screener-9 (BEDS-9): This modified version of the BEDS-7 (see Phase 1) incorporated two additional items suggested during expert review to capture DSM-5 diagnostic criteria: “During your episodes of overeating, how often did you eat more rapidly than usual?” and “During your episodes of overeating, how often did you eat until feeling uncomfortably full?”. Both were rated on the same 4-point Likert scale as items 3–7 of the original screener. These items were developed based on DSM-5 criteria for binge-eating episodes (i.e., eating more rapidly than usual and eating until uncomfortably full) to improve the representation of core features of the construct.
- Positive and Negative Affect Schedule [29–30]: Only the 10-item Negative Affect subscale was used, rated on a 5-point Likert scale. In Chilean samples, it has shown good reliability (α = 0.83) [30]; in the present study, ω = 0.84.
- Sociodemographic Questionnaire: As described in Phase 1.

#### Procedure

Participants were recruited between October 2023 and February 2025 through multiple channels, including (a) physical posters displayed in healthcare centers, (b) social media advertisements, and (c) referrals from healthcare professionals working with adults with overweight or obesity and recurrent binge eating episodes. All recruitment materials invited individuals to express interest in participating by completing an online registration form, which collected contact information only.

A total of 1,497 individuals registered their interest through this form. Of these, 719 individuals could not be reached despite repeated contact attempts and were excluded prior to screening. The remaining 778 individuals were successfully contacted and screened for eligibility by telephone.

Eligibility screening was conducted using predefined inclusion and exclusion criteria identical to those applied in Phase 1. Following screening, 330 individuals were excluded, and 448 were deemed eligible and invited to participate. Eligible participants provided informed consent electronically and were sent a link to complete the study questionnaires online via SurveyMonkey.

Of those invited, 13 participants did not complete the online questionnaires. The final analytical sample comprised 435 participants who completed the questionnaires and were included in the psychometric analyses. A participant flow diagram (Fig. 1) illustrates the recruitment, screening, and inclusion process.

Participants received a financial compensation of 10,000 Chilean pesos (approximately USD 10) for their participation. The study protocol was approved by the Ethics Committee of the Universidad Católica de Temuco. All participants provided informed consent prior to participation.

#### Data analysis plan

All analyses were conducted in RStudio version 2024.04.2 + 764 [31] using the *lavaan* package [32]. Outliers were removed using the Mahalanobis distance. Descriptive statistics were calculated for the sample, and total and average BEDS-7 scores were computed to classify participants into low- or high-risk groups.

To identify the optimal factor structure, confirmatory factor analyses (CFA) and exploratory structural equation modeling (ESEM) were performed with a Weighted Least Squares Mean and Variance Adjusted (WLSMV) estimator. Based on a parallel analysis, a two-factor solution (with correlated factors) was considered for both the BEDS-7 and BEDS-9. Six models were compared: (a–b) the original one-factor structures of the BEDS-7 and BEDS-9 (CFA), (c–d) two-factor structures of both versions (CFA), and (e–f) two-factor structures of both versions (ESEM).

Model fit was evaluated using CFI/TLI (≥ 0.90 acceptable, ≥ 0.95 excellent; 31) and RMSEA/SRMR (≤ 0.08 acceptable, ≤ 0.06 excellent) [33]. When models showed comparable fit, the most parsimonious was selected [34].

Measurement invariance across gender, physical activity, and BMI (overweight vs. obesity) was tested using multigroup CFA with four levels: configural, metric, scalar, and residual invariance [35]. Invariance was evaluated using standard thresholds (ΔCFI, ΔTLI < 0.010; ΔRMSEA < 0.015; ΔSRMR < 0.030) [36]. When needed, parameters identified by Lagrange multipliers were released [33].

Given the unequal gender distribution, analyses were replicated by comparing the full male group with a random female subsample of equal size. This procedure minimized bias from unbalanced groups and avoided overestimation of differences due to inflated confidence intervals.

Internal consistency was estimated using McDonald’s omega [37]. Items 1, 2, and 7 were excluded from psychometric analyses due to minimal variability under the inclusion criteria (100% endorsement of item 1; 97.7% for item 2; 97.2% “never” for item 7), which precluded their inclusion in latent variable modeling. Item 7 (self-induced vomiting), which functions as an exclusion indicator for bulimia nervosa in the original screener, was therefore not suitable for inclusion in factor analyses due to its restricted variability. Thus, factor and invariance models used items 3–6, plus the two DSM-5–based items from the BEDS-9.

Finally, to assess concurrent criterion validity, a structural equation model was estimated using WLSMV, with negative affect as the exogenous latent variable and the BEDS scale as the endogenous variable. Model fit was evaluated using the same criteria described above, and explained variance in the endogenous variable was calculated using the *R²* statistic.

## Results

### Results – phase 1

The translation and back-translation processes showed no major semantic discrepancies, and the expert review supported the linguistic and conceptual adequacy of the Spanish version, with only minor wording adjustments prior to pilot testing. The pilot evaluation did not reveal major comprehension difficulties. After this adaptation process, content validity was assessed using Aiken’s V coefficient along with 95% confidence intervals.

The results were: clarity: V = 0.86 (95% CI 0.69–0.94), coherence: V = 0.96 (95% CI 0.82–0.99), relevance: V = 0.86 (95% CI 0.69–0.94), and sufficiency: V = 0.68 (95% CI 0.49–0.82). Overall, the values were considered acceptable, except for sufficiency, which was lower. Judges emphasized the need to include missing DSM-5 diagnostic criteria—specifically eating more rapidly than usual and eating until feeling uncomfortably full. This recommendation was consistent with the lower V value for the sufficiency criterion. Consequently, two items were added, resulting in a 9-item version (BEDS-9; Table 3 shows the added items).

The decision to add these two items was theory-driven and aligned with principles of content validity [17], and is consistent with COSMIN standards [20], which emphasize the importance of content validity, including item relevance, comprehensiveness, and comprehensibility. Because the original BEDS-7 does not include these DSM-5 behavioral criteria, their inclusion allowed us to evaluate whether these central features of binge-eating phenomenology enhanced construct representation in the Chilean adaptation.

Regarding the item-level evaluation, all items showed acceptable Aiken’s V values (ranging from 0.71 to 1.00), except for item 7, which assesses the presence of self-induced vomiting. This item showed lower coherence (V = 0.57; 95% CI 0.25–0.84). This result was consistent with comments from some judges, who noted that the item addresses an exclusion criterion rather than a core symptom. However, given its essential role in differentiating BED from bulimia nervosa, it was retained in the final version.

**Table 3:** New items added to the original BEDS-7 to create the BEDS-9 version

Item No.	Item text (English)	Item text (Spanish)	Notes
8	During your episodes of excessive overeating, how often did you eat more rapidly than usual?	Durante sus episodios de comer en exceso, ¿con qué frecuencia comió más rápido de lo normal?	New item (expert suggestion)
9	During your episodes of excessive overeating, how often did you eat until feeling uncomfortably full?	Durante sus episodios de comer en exceso, ¿con qué frecuencia comió hasta sentirse desagradablemente lleno/a?	New item (expert suggestion)

### Results – phase 2

Among the total sample, 94.5% were classified as high risk for BED based on the BEDS-7, and 5.5% as low risk. Using the BEDS-9, 91.3% were classified as high risk and 8.7% as low risk. Descriptive statistics based on total scores are presented in Table 4. These were calculated excluding the self-induced vomiting item, as it is not part of the diagnostic criteria for BED; however, descriptive data including this item are also reported, given that some applications of the screener have incorporated it into the total score. As expected for a clinically enriched sample meeting recent binge-eating criteria, score distributions were skewed, indicating that findings primarily apply to high-symptom or clinically referred populations rather than community samples.

**Table 4:** Descriptive statistics Of BEDS-7 and BEDS-9 scores

Score	M (SD)	Minimum	Maximum
BEDS-7 (items 1–6)	10.49 (2.33)	3	14
BEDS-7 (items 1–7)	10.53 (2.35)	3	16
BEDS-9 (items 1–8)	14.63 (3.17)	5	20
BEDS-9 (items 1–9)	14.66 (3.19)	5	21

Factor analytic interpretations should be understood within the context of restricted variance and a clinically enriched sample.

Analyses showed that the one-factor BEDS-7 and BEDS-9 CFA models did not show adequate fit (see Table 5). The two-factor BEDS-7 ESEM model could not be estimated due to non-identification. In contrast, the two-factor BEDS-7 CFA model and the two-factor BEDS-9 CFA and ESEM models demonstrated excellent fit indices.

**Table 5:** Fit indices for alternative BEDS factor structures with CFA and ESEM approach

Model	χ^2^(df)	χ^2^/df	CFI	TLI	RMSEA (90% CI)	SRMR
BEDS-7 one-factor (CFA)	84.097 (2)	42.05	0.835	0.506	0.249 (0.205–0.0296)	0.116
BEDS-9 one-factor (CFA)	112.841 (9)	12.54	0.907	0.845	0.131 (0.110–0.153)	0.099
BEDS-7 two-factor (ESEM)	Not identified					
BEDS-9 two-factor (ESEM)	23.597 (4)	5.90	0.995	0.980	0.060 (0.038–0.085)	0.029
BEDS-7 two-factor (CFA)	1.489 (1)	1.49	1.000	0.998	0.014 (0.000–0.057)	0.008
BEDS-9 two-factor (CFA)	18.617 (8)	2.33	0.995	0.991	0.045 (0.018–0.071)	0.035

Closer inspection of each model (see Table 6) revealed that although the BEDS-9 models (both CFA and ESEM) exhibited acceptable factor loadings for the new proposed items (λ = 0.424 to 0.617), their internal consistency was questionable (ω_Global_ = 0.544, 95% CI [0.358–0.729]). In contrast, the BEDS-7 two-factor CFA model showed strong factor loadings (λ_F1_ = 0.811 and 0.596; λ_F2_ = 0.812 and 0.797), a moderate covariance between the factors (cov = 0.413), and adequate internal consistency (ω_Global_ = 0.706, CI 95% [0.658–0.755]; ω_F1_ = 0.668, CI 95% [0.596–0.742]; ω_F2_ = 0.788, CI 95% [0.738–0.837]). Consequently, this model was selected as the optimal factor structure for subsequent analyses. In this model, the first factor was labeled “loss of control” because its items reflect a lack of control during binge eating episodes (e.g., “How often did you feel like you had no control over your eating? ), whereas the second factor was labeled “emotional distress” since its items relate to unpleasant emotions such as guilt or shame resulting from uncontrolled eating (e.g., “How often were you embarrassed by how much you ate?”).

**Table 6:** Factor loadings for the alternative estimated models

Item	BEDS-7 one-factor (CFA)			BEDS-9 one-factor (CFA)			BEDS-9 two-factor (ESEM)				BEDS-7 two-factor (CFA)				BEDS-9 two-factor (CFA)			
	Λ	θ	*R* ^2^	λ	θ	*R* ^2^	λ_F1_	λ_F2_	θ	*R* ^2^	λ_F1_	λ_F2_	θ	*R* ^2^	λ_F1_	λ_F2_	θ	*R* ^2^
3	0.528 [0.438; 0.618]	0.721	0.279	0.615 [0.533; 0.698]	0.621	0.379	0.590 [0.475; 0.705]	0.122 [-0.005; 0.249]	0.588	0.412	0.811 [0.649; 0.972]	-	0.343	0.657	0.650 [0.565; 0.735]	-	0.578	0.422
4	0.459 [0.359; 0.559]	0.790	0.210	0.588 [0.506; 0.670]	0.654	0.346	0.692 [0.606; 0.778]	0.006 [0.000; 0.011]	0.518	0.482	0.596 [0.465; 0.728]	-	0.645	0.355	0.628 [0.548; 0.709]	-	0.605	0.395
5	0.675 [0.598; 0.752]	0.544	0.456	0.539 [0.453; 0.626]	0.709	0.291	-0.003 [-0.288; 0.282]	0.865 [0.527; 1.000]	0.253	0.747	-	0.812 [0.711; 0.913]	0.341	0.659	-	0.850 [0.757; 0.942]	0.742	0.258
6	0.674 [0.588; 0.760]	0.545	0.455	0.506 [0.414; 0.598]	0.744	0.256	0.013 [0.010; 0.015]	0.744 [0.530; 0.957]	0.440	0.560	-	0.797 [0.686; 0.908]	0.365	0.635	-	0.762 [0.667; 0.857]	0.619	0.381
8	-			0.496 [0.402; 0.590]	0.754	0.246	0.424 [0.292; 0.557]	0.136 [-0.015; 0.286]	0.763	0.237	-	-			0.508 [0.414; 0.602]	-	0.278	0.722
9	-			0.585 [0.501; 0.668]	0.658	0.342	0.535 [0.414; 0.655]	0.135 [0.002; 0.267]	0.647	0.353	-	-			0.617 [0.532; 0.703]	-	0.419	0.581

*λ* actor loading

*θ* residual variance

*R*^2^ proportion of item variance explained by the latent factor

After determining the optimal model, measurement invariance was assessed for gender, physical activity, and BMI (see Table 7). In the first step, configural invariance was tested, showing excellent fit for all grouping variables. In the second step, metric invariance was examined, yielding excellent fit without substantially worsening the configural model’s fit indices for the physical activity and BMI groups, indicating that the items have the same meaning across these groups. However, for gender, the change in fit indices was close to the cutoff criteria. Therefore, the analysis was replicated with a random female subsample of equal size to the male group. No substantial worsening was observed in this replication (ΔCFI = + 0.001; ΔTLI = + 0.020; ΔRMSEA = -0.032; ΔSRMR = + 0.005), so invariance testing continued with the next steps. In the third step, scalar invariance was tested, which showed excellent fit without substantially degrading the fit of the configural model for any of the grouping variables, indicating that the scale’s mean scores are comparable across groups. Finally, residual invariance was tested. This model showed excellent fit with no substantial differences from the configural model for the physical activity and BMI groups, implying that the indicators measure the same factor across these groups with equal precision. However, for gender, a considerable deterioration in fit was observed. Therefore, parameters were released to estimate a partial residual invariance model. Releasing the factor loading of item 4 yielded excellent fit indices with no substantial differences from the configural model.

**Table 7:** Fit indices for measurement invariance models of the two-factor BEDS-7

Model	χ^2^(df)	CFI	TLI	RMSEA (90% CI)	SRMR	ΔCFI	ΔTLI	ΔRMSEA
Grouping variable: gender								
Configural	4.774 (2)	0.998	0.990	0.036 (0.000–0.079)	0.013	-	-	-
Metric*	8.120 (4)	0.993	0.980	0.051 (0.000–0.101)	0.025	-0.005	-0.010	+ 0.015
Scalar	10.865 (6)	0.992	0.984	0.044 (0.000–0.086)	0.029	-0.006	-0.006	+ 0.008
Residual	16.710 (10)	0.989	0.986	0.042 (0.000–0.076)	0.037	-0.009	-0.004	+ 0.006
Grouping variable: physical activity								
Configural	7.352 (2)	1.000	1.014	0.046 (0.014–0.084)	0.014	-	-	-
Metric	4.022 (4)	1.000	1.021	0.004 (0.000–0.073)	0.016	0.000	+ 0.007	-0.042
Scalar	8.824 (6)	1.000	1.013	0.033 (0.000–0.075)	0.026	0.000	-0.001	-0.013
Residual	10.026 (10)	1.000	1.020	0.003 (0.000–0.054)	0.027	0.000	+ 0.006	-0.043
Grouping variable: BMI (overweight/obesity)								
Configural	1.931 (2)	1.000	1.031	0.000 (0.000–0.055)	0.007	-	-	-
Metric	1.458 (4)	1.000	1.034	0.000 (0.000–0.043)	0.007	0.000	+ 0.003	0.000
Scalar	4.121 (6)	1.000	1.027	0.000 (0.000–0.050)	0.016	0.000	-0.004	0.000
Residual	6.456 (10)	1.000	1.028	0.000 (0.000–0.038)	0.021	0.000	-0.003	0.000

Δ = Change in the fit indices; * = The analysis with subsamples indicated no substantial differences in fit indices

Finally, regarding concurrent criterion validity, the relationship between the BEDS-7 and negative affect was examined. The estimated structural model showed excellent fit indices (χ²[68] = 202.702, *p* < .001, CFI = 0.986, TLI = 0.981, RMSEA = 0.056, 90% CI [0.047 – 0.065], SRMR = 0.055), explaining 19.8% of the variance in loss of control and 24.9% in emotional distress. The standardized coefficients indicated that higher negative affect was associated with both greater loss of control (*β* = 0.445, 90% CI [0.329 – 0.562], *p* < .001) and greater emotional distress (*β* = 0.499, 90% CI [0.392 – 0.607], *p* < .001).

## Discussion

This study aimed to culturally adapt and evaluate the psychometric properties of the BEDS-7 in Chilean adults with overweight or obesity and recurrent binge-eating episodes. The findings indicate that the Chilean version shows adequate content validity, a clear factorial structure, acceptable internal consistency, factorial invariance across gender, nutritional status, and physical activity, and evidence of concurrent validity.

Although clarity, coherence, and relevance were adequate, low sufficiency led to the addition of two DSM-5–based items, producing a 9-item version (BEDS-9). This decision is consistent with COSMIN standards, which emphasize the importance of content validity—particularly item relevance, comprehensiveness, and comprehensibility—in the development and adaptation of measurement instruments [20]. This expanded version showed adequate factorial fit but low internal consistency, consistent with Herman et al. [15]. Thus, the BEDS-7 should be retained as the primary version of the instrument, while the BEDS-9 remains exploratory. It is important to note that the factor structure identified in this study is based on items with sufficient variability in a clinically enriched sample. Some items were not included due to near-zero variance. Therefore, the resulting structure should be interpreted as reflecting binge-eating symptomatology within a high-symptom population, rather than the full screening instrument.

Item 7 (self-induced vomiting) showed low variability and limited coherence because it reflects an exclusion criterion rather than a BED symptom. It was retained for its usefulness in distinguishing BED from bulimia nervosa [1], illustrating the tension between theoretical coherence and clinical utility described by Brod et al. [38]. Similar decisions appear in other studies [18, 22]. Its inclusion should depend on whether broad screening or diagnostic specificity is required. Future studies should also examine this item using diagnostic discrimination analyses to evaluate its contribution to accurate classification.

Regarding construct validity, unlike previous studies that conceptualize the BEDS-7 as a unidimensional scale [15, 18, 39], this study found a better fit for a two-factor model: one reflecting loss of control over eating and another reflecting the emotional distress that follows binge episodes. This distinction aligns with evidence from the BES, which identifies cognitive-affective and behavioral components [14, 22], suggesting that the scale captures both symptoms and underlying processes.

This pattern is consistent with theoretical distinctions between loss-of-control mechanisms and affective-cognitive distress described in emotion-related eating research [40, 41], supporting the interpretation of the BEDS-7 as reflecting symptom dimensions rather than a single global severity factor. These two factors may represent distinct yet interrelated psychopathological aspects of BED. However, given the limited number of items, this multidimensional structure should be interpreted with caution and requires replication in independent samples.

From a transdiagnostic perspective, they align with current models emphasizing the interaction between eating-related impulsivity, emotional dysregulation, and the use of food as a coping strategy [2, 42]. This perspective is also consistent with recent evidence indicating that DSM-5 diagnostic specifiers—particularly distress-related features—are associated with greater psychological burden and impaired quality of life, supporting the clinical relevance of the affective dimension identified in the present model [43]. Such differentiation may have clinical implications, as it could help guide more targeted interventions depending on whether distress or loss of control is more prominent. However, these dimensions should not be interpreted as independent diagnostic indicators, but rather as complementary aspects of binge-eating symptomatology within the context of a clinically enriched sample.

While gender invariance has been previously examined [18], this study found equivalence of the two-factor structure across gender, physical activity, and nutritional status. These findings indicate that group differences reflect true variation in BED symptomatology rather than differences in item interpretation [33], although comparisons across other groups (e.g., socioeconomic status) [44] highlight the need for further invariance testing.

In addition, the proposed structure for the BEDS showed evidence of concurrent criterion validity, as it was significantly associated with negative affect. This finding is consistent with previous literature documenting the central role of negative emotions in the onset and maintenance of binge-eating episodes [45].

A key pending challenge is validation of the BEDS-7 against a clinical gold standard, such as structured interviews including the Eating Disorder Examination (EDE) [46] and the Structured Clinical Interview for DSM-5 Disorders (SCID) [47]. Although some studies have used diagnostic algorithms derived from the instrument itself [21], its diagnostic accuracy remains unclear. Future studies should therefore assess sensitivity and specificity using structured interviews, with additional analyses such as ROC curves and Item Response Theory (IRT) [48–49] to refine cutoff points and examine item functioning. Importantly, classification as “at risk” based solely on BEDS-7 scores should be interpreted cautiously, as elevated scores may reflect current or past symptomatology rather than risk for future onset.

Research should also examine whether the two-factor structure reflects clinically meaningful subtypes, trajectories, or mechanisms within the broader BED spectrum and whether it replicates across diverse Spanish-speaking populations. In this context, the present study has notable strengths, as it provides one of the first country-specific psychometric evaluations of the BEDS-7 in Chile. Although the instrument had previously been included in a large multi-country study [18], that study did not report country-specific psychometric analyses or a linguistic adaptation process tailored to each context, and in the Chilean subsample only descriptive information was presented.

By addressing these gaps, the present study conducted a linguistic adaptation and evaluated the factorial structure, measurement invariance, internal consistency, and concurrent criterion validity of the BEDS specifically within the Chilean context, using a clinically enriched sample. Importantly, the identification of a two-factor structure in this sample further extends previous findings and suggests that the internal structure of the BEDS-7 may vary depending on the population and methodological approach.

Methodologically, the design was robust, evaluating multiple sources of validity and reliability and comparing alternative models. This approach allowed ruling out the need for cross-loadings to explain common variance [50]. Analyses were conducted in a clinically relevant sample, enhancing the instrument’s applicability in clinical contexts.

However, several limitations should be noted. The present study was not designed to provide a comprehensive evaluation of all psychometric properties of the instrument. Rather, it focused on selected properties appropriate for the scope and design of the study, including linguistic adaptation, internal structure, internal consistency, and concurrent criterion validity. In particular, test–retest reliability and convergent validity were not assessed in the present study. Although the sample size was adequate, participants met recent binge-eating criteria, which restricted response variability for some items. Thus, the findings apply primarily to high-symptom or clinically referred groups rather than to community-based screening.

The use of convenience and self-selected recruitment may also limit generalizability. In addition, BMI was calculated based on self-reported weight and height, which may introduce measurement bias and should be considered when interpreting the invariance results. Furthermore, the absence of a standardized clinical diagnostic criterion prevented estimating diagnostic sensitivity or determining an optimal cutoff. Future studies should incorporate structured interviews, include individuals across the BMI spectrum, and evaluate broader ranges of symptom severity to better determine diagnostic accuracy and generalizability.

## Conclusions

In conclusion, the Chilean version of the BEDS-7 demonstrated adequate psychometric properties in a clinically enriched sample of adults with overweight or obesity and recurrent binge-eating episodes.

These findings support its use for assessing key dimensions of binge-eating experiences, particularly loss of control and emotional distress, in clinical and research contexts.

However, its use as a screening tool and the establishment of cutoff scores require further investigation. Future research should examine diagnostic accuracy, test–retest reliability, and item functioning in more diverse populations.

## Data Availability

The data supporting the findings of this study are available from the corresponding author upon reasonable request.

## Abbreviations

BED: Binge eating disorder
DSM-5: Diagnostic and Statistical manual of mental disorders, fifth edition
BMI: Body mass index
EDE-Q: Eating disorder examination questionnaire
BES: Binge eating scale
BEDS-7: Binge eating disorder screener-7
ANID: National research and development agency
FONDECYT: National fund for scientific and technological development
BEDS-9: Binge eating disorder screener-9
PANAS: Positive and negative affect schedule
CFA: Confirmatory factor analyses
ESEM: Exploratory structural equation modeling
WLSMV: Weighted least squares mean and variance adjusted
